# Surfactant Phospholipid Kinetics in Ventilated Children after Therapeutic Surfactant Supplementation

**DOI:** 10.3390/ijms251910480

**Published:** 2024-09-29

**Authors:** Victoria M. Goss, Ahilanandan Dushianthan, Jenni McCorkell, Katy Morton, Kevin C. W. Goss, Michael J. Marsh, John V. Pappachan, Anthony D. Postle

**Affiliations:** 1 NIHR Biomedical Research Centre, University Hospital Southampton NHS Foundation Trust, Tremona Road, Southampton SO16 6YD, UK; v.m.goss@soton.ac.uk (V.M.G.); j.s.mccorkell@soton.ac.uk (J.M.); kathryn.morton@uhs.nhs.uk (K.M.); kevin.goss@uhs.nhs.uk (K.C.W.G.); jvp@soton.ac.uk (J.V.P.); 2 Clinical and Experimental Sciences, Faculty of Medicine, University of Southampton, Southampton SO16 6YD, UK; adp@soton.ac.uk; 3 Paediatric Intensive Care Unit, University Hospital Southampton NHS Foundation Trust, Tremona Road, Southampton SO16 6YD, UK; michaelmarsh@nhs.net

**Keywords:** paediatric, intensive care, ventilation, surfactant, phospholipids

## Abstract

Acute lung Injury leads to alterations in surfactant lipid composition and metabolism. Although several mechanisms contribute to dysregulated surfactant metabolism, studies investigating in vivo surfactant metabolism are limited. The aim of this study is to characterise surfactant phospholipid composition and flux utilising a stable isotope labelling technique in mechanically ventilated paediatric patients. Paediatric patients (<16 years of age) received 3.6 mg/kg intravenous *methyl*-D_9_-choline chloride followed by the endotracheal instillation of 100 mg/kg of exogenous surfactant after 24 h. Bronchioalveolar fluid samples were taken at baseline and 12, 24, 36, 48, 72 and 96 h after *methyl*-D_9_-choline infusion. Nine participants (median age of 48 days) were recruited. The primary phosphatidylcholine (PC) composition consisted of PC16:0/16:0 or DPPC (32.0 ± 4.5%). Surfactant supplementation resulted in a 30% increase in DPPC. *Methyl*-D_9_ PC enrichment was detected after 12 h and differed significantly between patients, suggesting variability in surfactant synthesis/secretion by the CDP-choline pathway. Peak enrichment was achieved (0.94 ± 0.15% of total PC) at 24 h after *methyl*-D_9_-choline infusion. There was a trend towards reduced enrichment with the duration of mechanical ventilation prior to study recruitment; however, this was not statistically significant (*p* = 0.19). In this study, we demonstrated the fractional molecular composition and turnover of surfactant phospholipids, which was highly variable between patients.

## 1. Introduction

Lung surfactant is essential for normal respiration to overcome surface tension generated at the alveolar air–liquid interface [1]. It is a complex mixture of lipids (90%) and proteins (10%) synthesised by the alveolar type II cells (AT-II) [2]. The lipid composition primarily consists of phospholipids, of which approximately 80% are phosphatidylcholine (PC), with the most surface-active component being dipalmitoyl phosphatidylcholine (DPPC) or PC16:0/16:0 at 40–60% in healthy individuals [3,4]. Both the total amount of functional surfactant and its composition are altered in lung injury [5,6,7,8]. However, while the optimum PC composition in healthy humans has been well defined, minimum physiological values for surfactant, particularly post surfactant supplement, have yet to be evaluated due to the difficulties in the accurate quantification of in vivo surfactant metabolism in humans [9].

In premature neonates, who typically have a homogeneous deficiency of surfactant material as a consequence of AT-II immaturity, treatment with exogenous surfactant therapy (EST) has demonstrable clinical benefits [10]. However, trials of EST in paediatric and adult patients with respiratory failure from Acute Respiratory Distress Syndrome (ARDS) failed to show any mortality benefits [11,12,13]. This is likely due to the clinical and pathological heterogeneity of lung injury, suggesting that stratification into trials by clinical indices was unsuccessful [6]. Therefore, phenotyping patients by surfactant composition and metabolism might allow for the stratification of this heterogeneous group of patients to provide guidance for the appropriate therapeutic treatment [14]. Improving the success of interventional trials to generate new treatment options for lung injury patients would bring substantial benefits, as current management is focused on protective ventilation strategies [15].

Studies dealing with the detailed in vivo analysis of surfactant metabolism are lacking in humans. Most isotope-labelled studies utilised stable isotope precursors such as U-^13^C-labelled fatty acids or deuterated water (D_2_O) to characterise disaturated PC (DSPC) kinetics by the osmium tetroxide-mediated oxidation of unsaturated PC from tracheal aspirated from ventilated patients with lung injury [16,17,18]. However, such studies lack specific information regarding detailed surfactant molecular distribution, and while the DSPC is used as a surrogate for DPPC (PC16:0/16:0), the labelled turnover measurements do not provide information on acyl-remodelling mechanisms.

Intravenous *methyl* D_9_-choline is a stable isotope that can be used as an alternative label to monitor PC kinetics [19]. The direct incorporation into PC via the CDP-choline pathway enables the rapid monitoring of the synthesis and turnover of all PC species. The incorporation into pulmonary surfactant PC in healthy adult volunteers, adult patients with acute respiratory distress syndrome and, more recently, in patients with COVID-19 has been demonstrated 24 h after intravenous administration [20]. *Methyl*-D_9_-choline incorporation into PC can be analysed from biological samples such as lung, plasma and urine using electrospray ionisation mass spectrometry (ESI-MS/MS) [21,22]. In this study, we explore the use of stable isotope labelling with *methyl*-D_9_-choline chloride and the ESI-MS/MS analytical methods to characterise surfactant composition and metabolism in paediatric patients with acute respiratory failure.

## 2. Results

### 2.1. Participant Demographics

There were nine paediatric subjects recruited with a mean age of 526 days (±436 days). All participants were mechanically ventilated for acute respiratory failure. Bronchiolitis related to respiratory syncytial virus (RSV) was the most common reason for acute respiratory failure. The median weight for the participants was 7.6 kg (±3.6 kg). All nine participants received a 100 mg/kg dose of Curosurf after 24 h of *methyl*-D_9_ choline infusion. The mean time from mechanical ventilation to *methyl*-D_9_ choline infusion was 66.7 h (±7.3 h). The demographic and outcome details of the participants are presented in Table 1. All patients survived their ICU illness.

### 2.2. Phosphatidylcholine (PC) Composition in Bronchoalveoalr Lavage Fluid (BALF)

**Figure 1:**
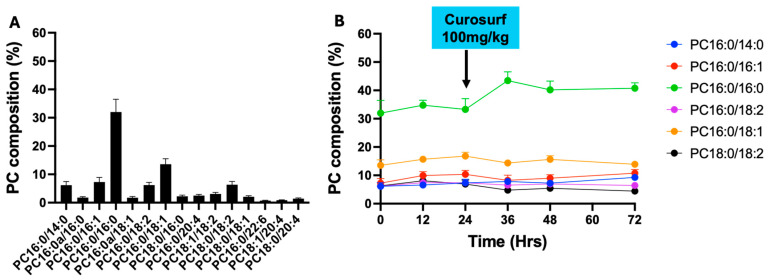
The bronchoalveolar lavage fluid phosphatidylcholine molecular composition at recruitment (T = 0) (**A**) and the fractional composition of major PC species over time (**B**). (N = 9 for time points 0 and 12, N = 8 for time points 24, 36, 48 and 72 h).

The primary surfactant PC species composition is detailed in Figure 1A. PC species with a fractional composition of >1% of total PC were included in the analysis. The 11 selected molecular PC species represented 77% of the total PC on the first BALF sampling at recruitment (T = 0). The PC composition at this first time point was characterised by PC16:0/16:0 (32.0 ± 4.5%), followed by PC 16:0/18:1 (13.2 ± 1.9%), PC16:0/16:1 (7.2 ± 1.6%), PC18:0/18:2 (6.3 ± 1.1%) and PC16:0/18:2 (6.2 ± 0.9%). The other disaturated PC species consisting of PC16:0/14:0 accounted for 6.1 ± 1.3%. Polyunsaturated and 1-alkyl-2 acyl PC species were rare (Figure 1A).

Most of the participants were extubated by 72 h, and consequently, the data beyond 72 h are only limited to one participant. Consequently, we have only presented the data up to 72 h after *methyl*-D_9_ choline infusion. The PC composition demonstrated little change over the time course. There was an increment in the DPPC composition hours following Curosurf supplementation from 33.3 ± 3.8% at 36 h to 43.5 ± 2.7% at 36 h (30% increase in composition). The fractional DPPC composition was relatively maintained until 72 h (Figure 1B).

### 2.3. The Total PC and Fractional PC16:0/16:0 Methyl-D_9_ Choline Enrichment

**Figure 2:**
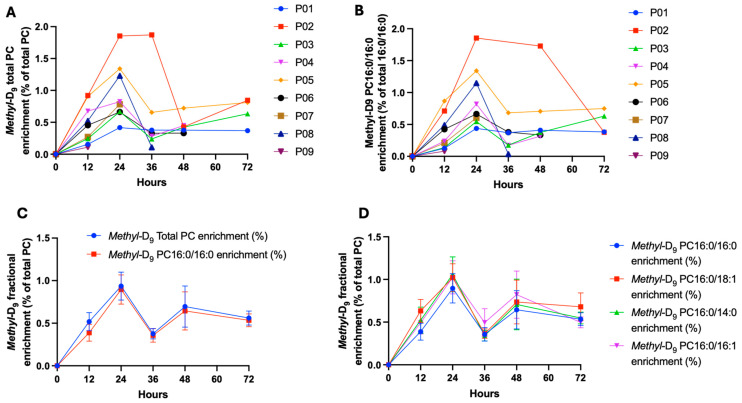
The total PC (**A**) and PC16:0/16:0 (**B**) *methyl*-D_9_ enrichment variability between patients, summary total PC and fractional PC enrichment (**C**) and comparison with other PC species (**D**). (N = 9 for time points 0 and 12, N = 8 for time points 24, 36, 48 and 72 h).

The total PC *methyl*-D_9_ enrichment and the fractional PC16:0/16:0 enrichments showed significant variability between patients, suggesting variability in the synthesis/secretion of surfactant PC synthesis by the CDP-choline pathway among individual patients (Figure 2A,B). The incorporation of *methyl*-D_9_ choline into total surfactant PC and PC16:0/16:0 was rapid, being, respectively, 0.52 ± 0.32% and 0.39 ± 0.30% enrichment at 12 h. Peak enrichment was at 24 h after *methyl*-D_9_ choline infusion (0.94 ± 0.16% and 0.90 ± 0.17% into total PC and PC16:0/16:0, respectively, Figure 2C). The subsequent decline in percentage enrichment after 24 h was likely due to the supplementation of Curosurf, as the enrichment is calculated relative to the unlabelled PC fraction, which is expected to increase following supplementation. The pattern of enrichment was similar between the surfactant-specific PC species (PC16:0/16:0, PC16:0/14:0 and PC16:0/16:1) and the other unsaturated species that are more characteristic of cell membranes (e.g., PC16:0/18:1) (Figure 2D).

### 2.4. Fractional Composition of Methyl-D_9_-Labelled PC

**Figure 3:**
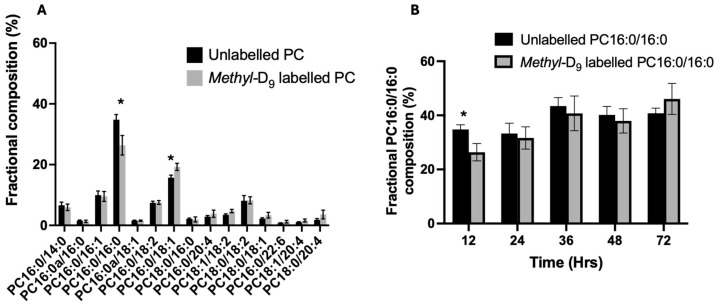
The fractional composition of unlabelled and *methyl*-D_9_-labelled PC at the first enrichment time point (T = 12 h) (**A**) and the compositional variation in unlabelled and *methyl*-D_9_-labelled PC16:0/16:0 over time (**B**). Data are presented as the mean and standard error of the mean. Student *t*-test analysis, * *p* < 0.05. (N = 9 for time point 12, N = 8 for time points 24, 36, 48 and 72 h).

Despite the similarities in the *methyl*-D_9_ enrichment between all surfactant PC species, the fractional composition of labelled PC was different from that of the endogenous PC composition. For instance, at the first labelled time point (T = 12 h), the composition of *methyl*-D_9_ labelled PC16:0/16:0 is much lower (26.4 ± 3.2%) than the endogenous composition (34.8 ±1.7%). However, the fractional composition of PC16:0/16:0 equilibrated with the endogenous composition by 24 h. In comparison, the *methyl*-D_9_-labelled PC16:0/18:1 was much higher than the endogenous composition at 12 h (19.3 ± 1.2% vs. 15.7 ± 0.85%) (Figure 3). This is likely to represent the acyl-remodelling mechanisms for the generation of PC16:0/16:0 from hydrolysis of PC16:0/18:1 with subsequent acylation.

### 2.5. Enrichment into BALF PC16:0/16:0 and Duration of Mechanical Ventilation Prior to Recruitment

**Figure 4:**
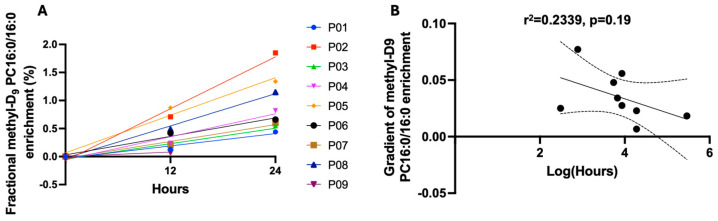
The gradient slope of the *methyl*-D_9_ PC16:0/16:0 enrichment for patients (N = 8) (**A**) and the correlation (95% confidence intervals) between the gradient and the log-transformed duration of mechanical ventilation prior to *methyl*-D_9_ choline infusion (**B**).

We investigated the possibility that the duration of mechanical ventilation before the study recruitment adversely affected the ability of alveolar type II cells to synthesise the surfactant. For this, we calculated the enrichment rate over the first 24 h, before Curosurf supplementation (Figure 4A). This rate demonstrated a wide variation between patients in surfactant PC 16:0/16:0 synthesis and was then correlated with the duration of mechanical ventilation before *methyl*-D_9_ choline infusion. Eight patients completed at least two sampling time points (12 and 24 h). Moreover, although most patients had their *methyl*-D_9_ choline infusion within 72 h of mechanical ventilation, one patient was intubated for a prolonged period (236 h). To avoid skewing, we long-transformed these data. There was a trend towards a negative association between the duration of mechanical ventilation prior to *methyl*-D_9_ choline infusion and PC16:0/16:0 enrichment, but this was not statistically significant (r^2^ = 2339, *p* = 0.19) (Figure 4B).

## 3. Discussion

In this study, we characterised the BALF surfactant PC composition and synthetic flux following *methyl*-D_9_-choline infusion in paediatric ventilated patients with acute respiratory failure. Although the fractional composition of DPPC was lower compared to a healthy population, the distribution was similar to previously published studies, where PC16:0/16:0 predominates the PC molecular composition. Following the supplementation of exogenous surfactant (Curosurf), there was an increment in the PC16:0/16:0 fractional composition by 30%. This was sustained for the subsequent time points. However, as most patients were extubated within 72 h, we could not characterise the BALF surfactant beyond this time. We used *methyl*-D_9_-choline to biochemically characterise the flux of PC secretion into the alveolar space. Our data demonstrate the biological diversity in children with lung injury, and the observed variation in the *methyl*-D_9_ enrichment reflects the differences in surfactant synthesis between patients. As far as we know, this is the first study to evaluate detailed surfactant molecular composition and dynamic PC synthesis in paediatric patients receiving exogenous surfactant.

PC is synthesised de novo by two distinct synthetic pathways. All cells use the CDP-choline pathway, while the phosphatidylethanolamine methyltransferase pathway (PEMT) is specific to hepatocytes and generates mostly polyunsaturated PC species [23,24]. The surfactant-specific DPPC is primarily synthesised by the CDP-choline pathway, and hence, isotope labelling with *methyl*-D_9_ choline enables the assessment of surfactant PC synthesis. However, a portion of DPPC is produced by acyl remodelling mechanisms, where unsaturated PC species are hydrolysed by phospholipase A_2_ to sn-1 palmitoyl lysophosphatidylcholine (lysoPC), which is then re-esterified with palmitoyl CoA catalysed by lysoPC acyltransferase to generate DPPC [25]. Our study is consistent with this paradigm, where at the earliest time point (T = 12), the composition of labelled PC had fractionally much higher unsaturated PC species and lower concentrations of DPPC. By 24 h, the newly synthesised PC fraction equilibrated with the endogenous composition, supporting the concept that surfactant DPPC is produced by both the CDP-choline pathway and complex acyl remodelling mechanisms.

While stable isotope labelling has been used extensively to study surfactant synthesis and metabolism from isolated AT-II cells and animal models, such studies in human injury in vivo are lacking. Moreover, most human studies used saturated PC (Sat-PC) as a surrogate marker to investigate DPPC composition and metabolism, which provides no details of molecular species distributions [16,17,18]. Our study investigated in more detail the composition and enrichment of the total BALF PC and, more specifically, the DPPC pool. We identified that the patterns of *methyl*-D_9_ enrichment into total PC and DPPC were similar. Moreover, we present the enrichment pattern for all major surfactant species for the first time, demonstrating that their secretion occurs at the same time as DPPC in this population. There was an apparent decline in the % enrichment after 24 h, which reflects the increase in the unlabelled PC fraction following exogenous Curosurf supplementation.

While exogenous surfactant in neonatal respiratory distress syndrome (NRDS) improves outcomes, the clinical effect of surfactant replacement in adult and paediatric populations with respiratory failure is not substantiated [11,12,13]. This is likely due to the lack of pre-characterisation of the potential population that may benefit from exogenous surfactant supplementation [26]. Clinical criteria such as the degree of hypoxemia are often used to identify patients who might benefit most from exogenous surfactant therapy [6], probably because of the current consensus definition of lung injury (ALI or the more severe ARDS) [27], do not necessarily predict diffuse alveolar damage nor the degree of disruption of alveolar surfactant composition or kinetics. The use of isotope labelling to define surfactant kinetic and compositional differences in a clinically relevant timeframe may enable future randomised controlled trials in predefined populations of children ventilated for ARDS. As our method could be reproduced using equipment and methods readily available in clinical Chemical Pathology laboratories, this methodology has potential for development as a translational tool for stratification into clinical trials instead of previously used clinical indices [28].

The variability in surfactant synthesis between patients may be due to several reasons: (1) The underlying pathology and the degree of alveolar epithelial injury resulting in the inability to synthesise and secrete surfactant adequately; (2) The availability of substrates such as fatty acids and diacylglycerol for the synthesis of surfactant PC; and (3) Variations in ventilation and the degree of alveolar stretch during mechanical ventilation. Lastly, surfactant metabolism is a complex process that can be influenced by multiple mechanisms that regulate phospholipid internalisation, transport, synthesis and secretion with variations in genetic and endogenous hormonal factors such as glucocorticoids and thyroxine availability [29]. However, such detailed molecular characterisation is lacking in human studies. Further larger real-world mechanistic studies are needed to explore detailed mechanisms of surfactant metabolism in vivo.

Although not statically significant, our data demonstrate that there may be a negative correlation with surfactant PC synthesis/secretion and the duration of mechanical ventilation, as suggested by Albert et al. [30]. While this must in part be due to the degree of lung injury and disease severity, it is possible that the process of mechanical ventilation itself can alter the metabolic pathway of surfactant production. Physiological stretch contributes to surfactant release and synthesis [31,32], and regular patterns of mechanical ventilation may disrupt this physiological cycle, which may have implications for adequate surfactant release. However, larger cohort studies are required to evaluate the effect of different durations and types of mechanical ventilation on surfactant secretion. Nevertheless, the demonstration of reduced surfactant turnover in patients ventilated for long periods has been demonstrated by other investigators [9,18].

This is a proof-of principle study and has several limitations, including the following: (1) we only recruited a small number of patients and were consequently not able to demonstrate any meaningful clinical outcomes, (2) as most patients were extubated by 72 h, we were not able to explore surfactant metabolism beyond this time, (3) we were not able to quantify the total surfactant pool size due to variability in the BALF surfactant recovery and (4) moreover, we did not collect specific granular data on mechanical ventilation variables, as this was highly dynamic depending on the degree of hypoxia and patients’ responses to treatment, with significant intra-day variability in ventilator modes and settings. However, patients were ventilated according to the lung protective strategy, with tidal volumes of 6–8 mL/kg predicted body weight and PEEP titrated according to the degree of hypoxia. Despite these limitations, in this study, for the first time, we were able to characterise and demonstrate the surfactant PC molecular composition and the mechanism and kinetics of PC synthesis in paediatric respiratory failure.

## 4. Materials and Methods

### 4.1. Ethics and Participants

The participants were children (<16-year-old) who were mechanically ventilated in the paediatric intensive care unit (PICU) for primary acute hypoxemic respiratory failure, defined as a PaO_2_/FiO_2_ ratio of <300 mmHg. Once inclusion criteria were met, informed consent was obtained from parents or guardians. The ethical approval of the study was provided by the Oxford C Local Research Ethics Committee (Number 07/H0606/125).

### 4.2. Study Procedures

**Figure 5:**
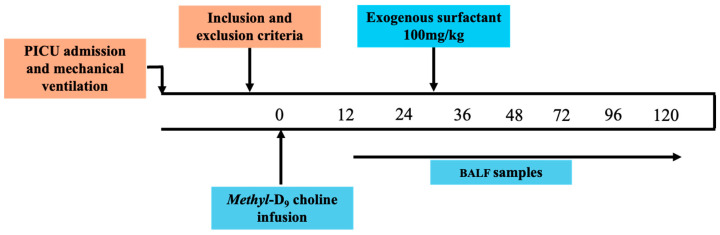
Study schedule. BALF, bronchoalveolar lavage fluid; PICU, paediatric intensive care unit.

After satisfying the inclusion criteria and after consent was obtained, the subjects received an intravenous infusion of 3.6 mg/kg of *methyl* D_9_ choline chloride (CK Isotopes Ltd, UK and Ireland) over 3 h. Except for one participant, most were recruited within 72 h of the initiation of mechanical ventilation. All participants received a single dose of an exogenous therapeutic surfactant preparation (Curosurf) (Chiesi Medical, UK) as a saline emulsion via endotracheal instillation at a dose of 100 mg/kg body weight. This was given 24 h after the infusion of the *methyl*-D_9_ choline chloride. This dose is commonly used for the treatment of neonatal respiratory distress syndrome in preterm infants and has been previously used safely in clinical trials of surfactant administration to adults and children with ARDS. A non-directed bronchoalveolar lavage (BALF) was performed at baseline and 12, 24, 36 and 48 h after the end of the *methyl*-D_9_-choline infusion and, subsequently, every 24 h until 120 h or extubation (Figure 5). Stabilisation fluid (200 µL 0.9% NaCl containing 10 µL of 20 g/L Butylated Hydroxytoluene) was added to all samples at the point of collection to prevent oxidation. The samples were centrifuged at 400× *g* for 15 min and the supernatant was stored at −80 °C.

### 4.3. Lipid Extraction

Lipids were extracted as previously described [33]. Briefly, 500 µL of BALF was made up to 800 µL with 0.9% NaCl, and 2 mL of methanol and then 1 mL of chloroform was added. Then, 10 nmoles dimyristoylphosphatidylcholine (PC14:0/14:0) was added to the sample as an internal standard before centrifugation at 1000× *g* for 10 min to pellet the protein. The supernatant was collected, and 1 mL of CHCl_3_ and H_2_O was added. The sample was centrifuged at 1000× *g* for 15 min to sharpen the biphase. Following isolation, the lipid-rich lower layer was dried under Nitrogen and stored at −80 °C.

### 4.4. Lipid Mass Spectrometry

**Figure 6:**
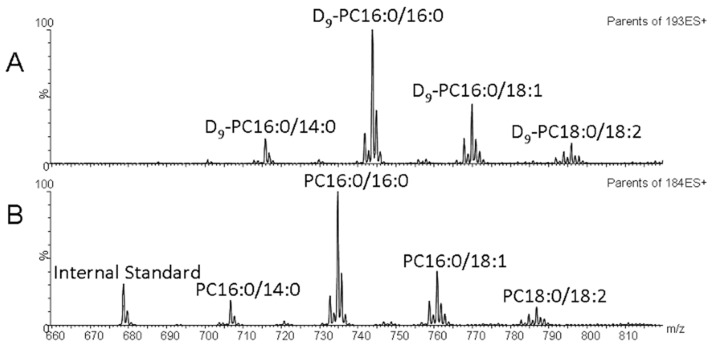
Example BAL Mass Spectrometry data. (**A**,**B**) Typical BAL direct infusion mass spectrum. (**A**) The newly synthesised *methyl*-D_9_-labelled PC species which have a diagnostic collision gas-induced product of *m*/*z* 193. (**B**) The endogenous PC species which have a diagnostic product of *m*/*z* 184.

Direct infusion Mass spectrometry was performed on a Xevo TQ (Waters). Collision-induced dissociation (CID) enabled specific headgroup fragmentation and identified the labelled and unlabelled PC composition. The PC composition and concentration were monitored by precursor ion scanning of the phosphorylcholine fragment ion at *m*/*z* 184.1 (P184), quantified against the PC14:0/14:0 internal standard. The enrichment of *methyl*-D_9_ choline in PC was determined based on the precursor ion scanning of *m*/*z* 193.1 (P193), according to the following formula: % enrichment = P193 × 100/(ΣP193 + Σ184), where ΣP184 and ΣP193 are the sums of abundances of unlabelled and labelled PC species, respectively. Examples of typical spectra for P184 and P193 are shown in Figure 6A,B. The data were collected and processed using MassLynx software version 4.1 (Waters). Raw data were analysed using an internally produced Microsoft Excel™-based macro after the isotopic correction.

### 4.5. Statistics

Data are presented as the means and Standard Error of Mean (SEM). The comparison between the groups is made by unpaired Student *T*-tests, and correlations between variables were carried out by Pearson Correlation Coefficient. Statistical significance was assumed when the *p* value was <0.05. We transformed to log scales when there was significant skewing. The data were analysed by GraphPad Prism version 10.0.0 for Windows, GraphPad Software, Boston, MA, USA.

## 5. Conclusions

The study results showed a wide range in the rate of PC synthesis, indicating differences in surfactant metabolism and turnover between subjects. Although there is a suggestion that the length of ventilation prior to recruitment influences surfactant synthesis, this was not statistically significant. Larger studies are needed to assess the surfactant metabolism following exogenous supplementation and how mechanical ventilation may influence surfactant synthesis in mechanically ventilated paediatric patients with acute respiratory failure.

## Figures and Tables

**Table 1:** Participant details and outcomes.

Variable	Patients (n = 9)
Age (days)	526 (±436)
Weight (kg)	7.6 (±3.6)
Curosurf dose (mg)	761 (±361)
Time from intubation to *methyl*-D_9_ choline infusion (hours)	66.7 (±7.3)
PaO_2_/FiO_2_ ratio at enrolment (mmHg)	166 (±19)
Diagnosis at admission	
RSV Bronchiolitis	n = 4
Bronchiolitis (other)	n = 3
Pneumonia	n = 1
Other	n = 1
Duration of mechanical ventilation (days)	8.3 (±0.5)
ECMO (n, %)	1 (11.1%)
Survival (n, %)	9 (100%)

Data are presented as numbers (%) and the median and interquartile range (IQR).

## Data Availability

The data that support the findings of this study are available upon request.
